# History of a Fatal Case of Hydrocephalus

**Published:** 1808-01

**Authors:** 

**Affiliations:** Kingston, Jamaica


*History of a fatal Case of Hydrocephalus. By Dr.* HARRIS, *of Kingston, Jamaica, in a Letter to Dr.* FARQUHAR, *dated July* 30, 1805.

NOTHING tends so much to the advancement of medical knowledge as a faithful record of cases. It is not only profitable to the recorder, but it behoves him to publish his practice for general advantage. A candid practitioner will never be afraid to have his endeavours for the benefit of his fellow creatures canvassed by the public eye; because, self not being his prevailing principle, he is conscious of having exerted himself both for the good of his patient and profession.

M. K. H, in her twenty-sixth month, a healthy child, of a very lively disposition, and uncommon sagacity, on the 20th of October, 1802, discovered symptoms of indisposition, from being more inactive than usual, but had no particular complaint. She took two grains of calomel at bed time, and was carried to the country next morning to stay a few days. She did not discover her usual desire for breakfast, and brought up some milk she had taken, in a curdled state, some time after. Her mother gave her some magnesia, which operated once during the next night; and brought her to town on the morning of the 22d. I found her dull and heavy; but she had no symptoms of fever, farther than a small degree of partial warmth in the palms of her hands and the soles of her feet. As she was about cutting her last jaw teeth, and complained of them, the gum being swelled, I scarified it. She then took an emetic, which brought up some green undigested stuff, but it did not carry off the drowsiness. At night she took an antimonial, and passed a restless night; with an incessant short cough. This gave the idea of cold and indigestion being the cause of indisposition.

23d. She took castor oil, which operated once, and brought away some ascarides. The antimonial was repeated at night.

24th. She took rhubarb and calomel, which came up with some phlegm. At night she took three grains of calomel, which operated three times before morning.

25th. The oil was repeated, which produced one motion. She passed this day in a kind of unwillingness to do any thing but slumber: but was much troubled with the cough. Her skin was cool but dry, and she was put into into a tepid bath without any visible good effect; but seemed rather to oppress her breathing while she was in it.

26th. A restless night. The cough was incessant to-day; but did not call forth much affection when it attacked her. It seemed to proceed from the stomach; and, keeping her from sleep the whole night, an emetic of ipecacuan was given her on the 27th, which lengthened the intervals of coughing the succeeding night.

28th. The stupor increasing and the pulse varying, tho' not above seventy in a minute, I began to be much alarmed at her situation; as she evidently grew weaker and weaker. My feelings were now so acute as to stagger my judgment: and I requested the assistance of some medical friends. Her bowels having been torpid since the last purgative, and suspecting worms, some assafœtida glysters were advised and administered at different times through the day; and a blister was applied between the shoulders to relieve the head. Towards evening she grew so alarmingly ill from the cough, unequal breathing, and puffing of the belly, that five grains of calomel were administered, and followed up with two large spoonfuls of castor oil, some hours after, at different times. Stimulating cataplasms were likewise applied to the feet. The blisters were dressed about the time the cataplasms were removed, and she had a respite of two or three hours of rest. During the night, she had a large fæcal discharge with the fœtid glyster. The cough was somewhat relieved; but the pulse continued variable and unnaturally slow. It was remarkable that, notwithstanding the stimuli of the blisters, the cataplasms, and the fœtid glyster, besides several glasses of pure wine, there was no excitement in the arterial system, or heat on the skin. These symptoms, although ambiguous, might still be considered as proceeding from worms.

29th. The stupor continued although the pulse was not quite so variable: it was still however below the natural standard. A powder of rub. ferri, pulv. rhei and calom. ana gr. v. was given, and, about two hours after, there was such a conflict between the disease and medicine, as was very alarming. She had a large stool, which so sunk the powers of life as wine and vol. frictions could scarcely recall. A blister was now applied to the epigastric region, and nourishment and wine administered frequently. The pulse soon became more regular, but still below nature; the skin cool. In the evening she had a large slimy stool; and had some hours of natural rest after having the blisters dressed. About ten o'clock she grew restless and uneasy, with a slight return of cough. Two grains and a half of calomel and five of rhubarb were now given, and to be repeated towards morning if necessary. The first dose created some sickness at the stomach, which, in her very weak state, distressed her much: a glyster was therefore thrown up, which procured another slimy stool, and in two or three hours she had a little rest.

Towards five o'clock in the morning of the 30th, she had another stool, dark and slimy. There was now a greater degree of warmth in her hands and feet than had been felt from the beginning: pulse the same as before. By moaning and sighing, she expressed great anxiety and debility. About eight A. M. her blisters were dressed, and tepid sponging with vinegar and water was used, which produced a little quietness and rest. Beef tea, with table salt, sago, gruel, and wine, were freely administered. Soon after she had another slimy dark stool. Symptoms now grew so alarming as to create a strong suspicion of hydrocephalus internus. The shutting of her eyes, which was before attributed to coma, was now plainly discovered to be owing to the disagreeable effect of light. There were added to this symptom a perpetual sighing and moaning, with long and interrupted respirations, an incessant motion in the left arm and leg, with frequent pointing to the head, and a much greater irregularity of the pulse than had been yet discovered. She was likewise quite inattentive to every thing that was said to her. To mitigate these symptoms for the present, five drops of laudanum and ten of æther were given to her, after letting her sit in the semicupium for a few minutes. During the effect of the opiate her head was shaved, and a large blister applied, after rubbing the scalp well with camphorated rum. Mercurial frictions were now vigorously adopted, and five grains of calomel were given about nine o'clock in the evening. From the opiate and friction, the pulse was now more equal and full than it ever had been; and she remained pretty quiet and easy until ten o'clock; when another strong friction was performed, and an antimonial opiate (twenty drops of the wine to ten of the tincture) was administered. This had no effect until past eleven, when she fell asleep. She rested until five o'clock, but moaned and sighed very frequently.

30th. After day-light, it was discovered she had lost the use of the upper eye-lid; but opened the other as she had done for several days before, only to shut it immediately ately against the effect of light. During this afflicting scene, the poor little sufferer astonished every beholder in the discovery she made respecting the affected eye, by placing her fingers on the right eye, while she stretched open the left; which she could not accomplish before she had shut the right: The motion of the left arm and foot remained the same. Had no extraordinary motion in the right. Was perfectly sensible. The mercurial friction was repeated this morning, and a stimulating glyster with Epsom salt was administered, which gave her two large, glareous, brown motions; after which she opened her eyes better. The pulse was fuller and more frequent, with more heat on the extremities. The pupils, particularly the left, much dilated for the first time since the commencement of her illness. The glyster was repeated about ten o'clock A. M. from which she had another discoloured motion. A solution of the Epsom salt in a strong beverage equally saturated with sugar and lime juice, was now given by a spoonful at a time every half hour, until she had an healthy-looking stool, about two o'clock P. M. and another of the same kind within an hour after. She was now more observant; called for what she wanted, and was also more tranquil. A large blister had been renewed to the head, which about this time shewed signs of discharging; it was suffered to remain on till night. Her nourishment, gruel and beef tea made palatable with table salt. Her pulse was regular and full, her skin temperate, but the pupils still dilated. She had several stools of nothing but the nourishment she took; she became a little restless towards bed-time; and she took three drops of laudanum and six of antimonial wine.

November 1. Slept without moaning, and the blister discharged plentifully in the night. There was little or no affection of the left arm and leg; but the left eye was still half closed, and both pupils enlarged. The mercurial friction was continued, and an infusion of bark was given. She lay quite passive all day, took nourishment when given, and had two small natural stools. Towards evening she seemed much inclined to doze, and sighed a good deal. The pulse was rather tending to variation from a seeming retardation of one pulsation now and then. A blister was applied to the occiput and neck after a mercurial friction. These symptoms increased, with a febrile heat and interrupted suspirations, which seemed at times to be almost entirely suspended. These were attended with deep sighs and heavy groans. The right arm and leg leg became now affected in the same way as the left was some days ago, with alternate flushings of the face. The antimonial opiate (twenty drops to ten) was given about 11 o'clock at night, which by degrees subdued all these, apparently indomitable, symptoms into a sleep, which brought her to the

2d. With a regular pulse at 116. She has not spoken since yesterday noon; and that was very faintly, although she has been perfectly sensible; for when she heard the sobs of those around her, when they thought her dying, she opened her eyes, and looked steadily at them for some time. The powers of life seem wavering; the hands and feet continue hot, while every other part is temperate. Though the pupils seem somewhat less, the eyes have lost their lustre considerably. There is a rattling phlegm in her throat when she coughs, which is now but seldom. There were ordered two grains of pulv. scillæ, one of calom. and six drops of the tinct. digitalis, with a mercurial friction. At ten o'clock the pulse rose to 120. Six drops more of the tincture. In less than one hour the pulse fell to 100—full—somewhat variable. Lies quite passive, and sighs frequently. Pulse rose again in ten minutes to 120. At twelve o'clock there was a general moisture. She had passed some urine. The dose was repeated, as at ten. At two o'clock, P. M. the pulse was at 135. She had passed more urine, and was tranquil. Eight grains of tinct. dig. were given in beef-tea, and another mercurial friction applied. Had a large dark stool at half past three P. M.; pulse 135. A purging glyster gave her a large motion, and she sunk into an easy sleep immediately; during which the pulse sunk to 112, at five o'clock P. M. At six o'clock, pulse 120. Repeated the calomel and squills, with ten drops of the tinct. digitalis. At seven o'clock, the pulse 103. Slept, after the fatigue of dressing her blisters, for two hours. A mercurial friction about eight o'clock. She grew a little restless soon after; pulse 130. Had two pukes, which might have been occasioned by increasing the squills to four grains. She was easy after it, and fell asleep with the pulse at 116; and slept without interruption, but from nourishment, till almost 12 o'clock, when the symptoms which hitherto required the antimonial opiate began to appear, with a small pulse at 136, and alternate flushings. This hitherto powerful medicine was given in the same proportion as last night.

3d. She rested tolerably well, and was awakened to take nourishment. No flushing to-day; pulse 132; skin warm; warm; had urined once; medicines continued. At ten A. M. she was so sick at stomach, as to have nearly fainted; pulse 120; a little toast and water settled her stomach, and she enjoyed some rest. She is averse to nourishment to-day, owing, probably, to her mouth being affected by the mercury. Had a slimy stool about one o'clock P. M. more general heat than usual; pulse 125. There is no strabismus; but the globes are directed more to the left than usual. The pupils seem somewhat contracted, and sensible of the light. About five o'clock P. M. she had a large loose stool, which contained several leaves of green resembling spinach, called in Jamaica caliloo, undigested. This was one article of which she ate on the 19th of October. Took very little nourishment to-day; had another small stool at nine o'clock; attempted to give her the bark infusion with spiritus minder. but she could hardly swallow; breathes with some difficulty; pulse 130, regular. At half past ten o'clock pulse 145; more difficult breathing and swallowing. At eleven, pulse 150. Administered a bark glyster, with the antimonial. In ten minutes after the pulse was 130, and breathing better. Two other attempts were made to throw up glysters; but they were ejected immediately. There have been several contractions of the ankle and wrist of the left side to-day. The pulse was fluctuating between 120 and 130. The antimonial was continued.

4th. The pulse 112 at five o'clock A. M. with an open skin and easy respiration. At six o'clock she had some nervous twitches in the muscles of the left cheek, while the arm and leg of that side were drawn up involuntarily. At these periods the pulse varied considerably. A stool gave some respite to symptoms; a purging glyster was thrown up, without any effect but flatulence. The convulsive affections of the extremities and left cheek continued to recur, with increase every five minutes through the day, with additional quickness of pulse, even to 170.

My medical friends had taken leave since yesterday, and I was in a state of mind not to be described: however, it suggested cupping. But, unfortunately, glasses could not be procured. I then resorted to musk, merely to smooth the rugged path to death by convulsions. The musk and antimonial were continued the whole day, during the short intervals of the convulsions; and about six o'clock P. M. they were less frequent, and she shewed some disposition to sleep. I therefore resorted to the antimonial opiate once more, in proportion of six to twelve drops of of the laudanum and antimonial. She was quiet for more than an hour, had no spasms, and turned herself with more freedom.

Nourishment and the drops were instilled through her teeth, which the weak powers of deglutition now and then sent into the stomach. The pulse averaged 150. At the same time as usual, the turn of the night, she grew worse, sighed and moaned; when twelve drops of antimonial wine and six of laudanum were given, which prevented a return of the convulsions. She had some slumbers, and between whiles two or three injections were given, which relieved her of considerable flatulence and some slime from the bowels. There were opiate frictions applied to the belly.

5th. Pulse 152, equal; a very small degree of contraction in the pupil. She moaned a good deal, as if from uneasiness in her bowels. An asafœtida glyster was given, which was soon discharged with a considerable yellow tinge. Another was thrown up, warmer, which she retained about half an hour. Considerable symptoms of uneasiness led to the application of warm cloths to her belly. The glyster was discharged with the same appearance as the former. She was somewhat relieved, but moaned considerably. A tea spoonful of gin in warm toddy was given, and another fœtid glyster with ten drops of laudanum. At two o'clock P. M. pulse 148, regular and stronger; still moaning; took calomel grs. iij. aloes grs. ij. At four o'clock she passed the last glyster with some greenish fæces. She was quiet, and slept till past five; deglutition better; took some nourishment; grew uneasy again between eight and nine o'clock, which was probably occasioned by the purgatives she had taken: however, the friction for some time of a hand over the abdomen, composed her again. Finding that the friction by a hand alone gave ease, the size of a nutmeg of mercurial ointment was rubbed in all over the surface of the abdomen in the direction of the colon, which, in about twenty minutes, procured a large stool of brown excrement and glareous matter. She took some nourishment and went to sleep.

The hands and feet, which have hitherto always much exceeded the temperature of the body, have this day shewn some signs of amendment, by being equally temperate with the rest of the body. Pulse 160; she grew restless at the usual period of midnight; when the left foot grew hot, with some approach to spasm on that side. An injection brought away another stool, and she was easier before one o'clock o'clock in the morning. There was observed a slight degree of inflammation on the sclerotica of the left eye during this day. She had but little sleep; pulse 170; a coldness of both feet now took place, extending soon as high as the knees. In half an hour this was succeeded with heat in the left foot, and stiffness in the left arm, accompanied with great restlessness. Musk was given in her nourishment, which was swallowed without difficulty.

At one o'clock in the morning of the 6th, all the left side was affected by spasm, which was relieved by a glyster, and she lay composed till past two, when she was attacked in the same manner, and another glyster thrown up. This remained till about three, when she passed it with some bloody mucus. From this time she was lulled till half past four; when, on the return of great restlessness, the antimonial opiate was given as last night. This procured about an hour's respite; after which there was a general tendency to spasm, with much anxiety, tossing, and coldness of the feet; to which frictions and warm flannels were applied. At eleven o'clock the pulse was 200; her blisters were dressed; not much restlessness now, but suspirations very frequent; no spasms have appeared lately; the mercurial frictions were continued; her belly, legs and feet were fomented for an hour or two with warm bark decoction, to incite the return of heat, but the powers of life were not now to be recalled. She expired at nine o'clock in the evening.

Upon opening the head, the blood-vessels appeared much distended, but there was no extravasation or serous effusion between the membranes. Upon removing the membranes, the fulness of the vessels on the surface of the brain, exhibited their ramifications in a most beautiful manner; and upon cutting through the corpus callosum, there were found between three and four ounces of a pure limpid fluid. This was sufficient to account for the death of the child without going further.

From these appearances we may reasonably presume, that had the disease been suspected in its first stage, there might have been some probability of success from lessening the quantity of blood in the head, either by leeches, arteriotomy, or cupping, and by the early use of blisters and setons, accompanied by the powerful effects of mercury.

Two of these remedies were vigorously persisted in; but, alas! at too late a period, although they were applied two days before any dilatation of the pupil took place; even even their late use evinced their power on the disease in a very striking manner.

Improper indulgencies in diet may be considered as pre-disposing causes of hydrocephalus internus; for an healthy state of the stomach is absolutely necessary to a proper discharge of all the animal functions. It is true, we are not sufficiently acquainted with the functions of the brain, to account for the production of its diseases; but we know that its structure is so wonderfully fine, as to be easily affected by the derangement of the fountain of health, the stomach; especially if other causes operate at the same time; as was the case in the present instance.— Much might be said on this part of the subject, but it is sufficient to remark, that we are too apt, if a child is generally healthy, and endowed with good digestive organs, to let affection outrun judgment, in giving what ought to be denied to its utmost importunities. Such gratifications are fraught with disease. But, besides the benefit that a resolute conduct in this respect would afford to health, a proper restraint is wholesome in a moral sense; in teaching children obedience, and habituating them to an early self-denial, which will arm them against many future mortifications in life.

Before and on the 19th of October, there was neither "heaviness of the eyes, nor diminution of nervous energy, nor drowsiness," nor, in short, any of the other symptoms which distinguish hydrocephalus, or indeed any other complaint. On the contrary, blooming health prevailed in an eminent degree; so as to be particularly remarked by the family, with whom that day was spent in the country. Being thus lulled, as it were, into security, the child was caressed, and suffered to eat perhaps of improper things at a much later hour than usual. She was brought home late, and it was a rainy evening. Under such circumstances, the treatment during the former period of her illness was certainly rational, though unfortunate. When the symptoms became more characteristic of hydrocephalus internus, it occurred to me that she had a fall some five or six weeks before; which, having left no signs of lesion whatever, had been entirely forgotten.— None of her numerous connections had been ever subject to the disease; so that there can be no room to lay this disease to constitution. Her head was rather small, with a very compact bone.

Here a query naturally arises, viz. Could water collect in the ventricles of the brain, to the quantity of more than three three ounces in so short a time as eighteen days? Or could the fall be the cause of the disease, without shewing some symptoms of it before the 20th of October, which was the first day of any illness? In the first instance, obstructed perspiration from cold, when that evacuation was copious after a full meal, might probably have laid the foundation of the disease on the evening of the 19th; when the further aid of deranged stomach and bowels might have contributed towards its progress, by destroying the balance between the exhalents and the absorbents. And, if it be true, as the sagacious Rowley observes, that vomits contribute much towards producing the dropsy of the ventricles of the brain, the accumulation must have been accelerated in this case by the treatment at first. If a fall was the primary cause, the high health which the child enjoyed after the accident must have retarded the formation of the disease, until the above morbid actions took place; and these would probably seize on that delicate part which had before suffered concussion.

Since the termination of this case, I have perused Dr. Rowley's ingenious treatise on Hydrocephalus Membranarum with much pleasure and information. He says, that it generally precedes hydrocephalus internus. The Doctor mentions several symptoms that did not occur in the above case; but as the drowsiness and listlessness to action occurred in the beginning, as he mentions, it may be asked, could the dropsy of the membranes be changed into that of the ventricles, without having any marks of the first disease to be seen on dissection? Or may not drowsiness or listlessness to action, accompany a morbid state of the stomach and bowels? or a case of worms? It appears from the above case,

1st. That diseased action of the stomach and bowels, and obstructed perspiration, may prove the proximate cause of Hydrocephalus Internus.

2dly. That Hydrocephalus Internus may be occasioned by concussion.

3dly. That Hydrocephalus Internus did not exist before, but must have commenced on or after the 19th of October; when, the powers of health being abstracted, any injury formerly sustained, might have commenced its action on the brain.

Although Hydrocephalus Internus has been very correctly described by several eminent authors, it appears that the disease has been too often overlooked in practice, on account of the similarity of symptoms with other diseases eases incident to children. I must confess that I have considered it as a rare disease, until the fatal termination of this case, which came so home to my feelings as to induce me to look back with regret on several cases, in which I might have overlooked the disease.

Happy would it be for mankind, if each individual would profit by the consideration of the errors he has committed, either in theory or practice. Severe as the sacrifice may be considered to the feelings of human nature, I should be happy could I be assured that the record of a case which has deprived me of the endearments of a beloved and most promising child, may be the means of information to myself, or comfort to other parents, in warding off the fatal stroke from their offspring!

I cannot but lament the general repugnance that prevails here among medical men, to inspect bodies after death; as it is the only true source of information, and ought to be an invariable rule with every practitioner, when he cannot clearly ascertain the cause of death, strongly to solicit for permission to search for it by dissection. Such general conduct would evince to the world that the love of information and improvement in the art of preserving life was of much higher consideration in the minds of medical men, than the accumulation of wealth.

As all the means, to the best of my knowledge, which have been recommended by others, have been unsuccessfully tried in the above case; it only remains to say, that this fatal disease may be much easier prevented than cured. Indeed, if means of prevention were more studied by practitioners and parents, there would be less occasion to prescribe remedies for the cure of infantile disorders in general.

The precepts laid down by Dr. Rowley, respecting children, at the end of the work already mentioned, are judiciously adapted to all capacities, and ought to be generally studied.

Scarifying the gums has been objected to by some practitioners, under a false notion of producing a cicatrix firm enough to impede the progress of dentition: but when Dr. Rowley forbids this operation to be performed "too early," his caution cannot be considered as favourable to that opinion; for experience confirms that, when the swelling and inflammation evidently shew that one or more teeth are at hand, the only mode of giving instantaneous relief from any violent symptoms threatening convulsions is, not only only to open, but to take off a portion of the gum down to the advancing teeth.

Opinions may sometimes lead to improvement; but, when experience has established a fact, opinion should yield; because in founding a practice on this, without being sanctioned by the other, we certainly lead ourselves and others into much dangerous error. The experience I have had in the good effects of mercurial preparations against malignant fever, and in diseases of children, induces me, without hesitation, (whatever violent condemnations have been uttered against them,) to recommend the practice in the early stages of those, (if neglected) most dangerous affections in both periods of life.

Both malignant fever and hydrocephalus internus, in their advanced stages, seem to resist the power of mercury in a similar degree; but both, I presume, may be prevented or obviated in their fatality, by the powerful agency of this medicine on the system, before the torpor, which these diseases create, takes place too firmly on the animal functions, and thereby renders its operation inert. In the same manner therefore as I have advised, (see Medical and Physical Journal, volume ix) all who may be obnoxious to that fever to prepare against it, do I now recommend calomel to be frequently given to children, to guard them against hydrocephalus internus: for it is to be feared that this dire disease is more fatal to them than is generally suspected; and that, from the similarity of symptoms, the cause of death is too often attributed to worms or some other imaginary disease.

I have only to add that, as I have always kept an eye of suspicion on every fever, attacking a stranger to this climate, ever since the commencement of this scourge to the West Indies in 1794; I have found that a prompt and vigorous practice at the beginning had a decided advantage, when malignity, although not at first suspected, supervened in a few days. So it may be with hydrocephalus, if symptoms are in the least ambiguous; for, if the case should be either worms or infarcted bowels, the mercurial treatment recommended in hydrocephalus is equally applicable to these. Evacuations from the head will depend upon circumstances attending the state of the sensorium.

